# Volatile Emissions from *Mycobacterium avium* subsp. *paratuberculosis* Mirror Bacterial Growth and Enable Distinction of Different Strains

**DOI:** 10.1371/journal.pone.0076868

**Published:** 2013-10-08

**Authors:** Phillip Trefz, Heike Koehler, Klaus Klepik, Petra Moebius, Petra Reinhold, Jochen K. Schubert, Wolfram Miekisch

**Affiliations:** 1 Department of Anaesthesia and Intensive Care, University of Rostock, Rostock, Germany; 2 Friedrich-Loeffler-Institut, Federal Research Institute for Animal Health, Jena, Germany; Colorado State University, United States of America

## Abstract

Control of paratuberculosis in livestock is hampered by the low sensitivity of established direct and indirect diagnostic methods. Like other bacteria, *Mycobacterium avium* subsp. *paratuberculosis* (MAP) emits volatile organic compounds (VOCs). Differences of VOC patterns in breath and feces of infected and not infected animals were described in first pilot experiments but detailed information on potential marker substances is missing. This study was intended to look for characteristic volatile substances in the headspace of cultures of different MAP strains and to find out how the emission of VOCs was affected by density of bacterial growth. One laboratory adapted and four field strains, three of MAP C-type and one MAP S-type were cultivated on Herrold’s egg yolk medium in dilutions of 10^-0^, 10^-2^, 10^-4^ and 10^-6^. Volatile substances were pre-concentrated from the headspace over the MAP cultures by means of Solid Phase Micro Extraction (SPME), thermally desorbed from the SPME fibers and separated and identified by means of GC-MS. Out of the large number of compounds found in the headspace over MAP cultures, 34 volatile marker substances could be identified as potential biomarkers for growth and metabolic activity. All five MAP strains could clearly be distinguished from blank culture media by means of emission patterns based on these 34 substances. In addition, patterns of volatiles emitted by the reference strain were significantly different from the field strains. Headspace concentrations of 2-ethylfuran, 2-methylfuran, 3-methylfuran, 2-pentylfuran, ethyl acetate, 1-methyl-1-H-pyrrole and dimethyldisulfide varied with density of bacterial growth. Analysis of VOCs emitted from mycobacterial cultures can be used to identify bacterial growth and, in addition, to differentiate between different bacterial strains. VOC emission patterns may be used to approximate bacterial growth density. In a perspective volatile marker substances could be used to diagnose MAP infections in animals and to identify different bacterial strains and origins.

## Introduction

Paratuberculosis (*paraTB*), a chronic progressive enteritis of domestic and wild ruminants, is prevalent in all countries with intensive dairy, beef or sheep industry. In the U.S., e.g. decreasing productivity of dairy and beef cattle and contaminated milk and cheese are estimated to cause annual losses from $200 million up to $250 million. Furthermore, there is some evidence, that MAP, the causative agent of *paraTB*, could be a cofactor in chronic inflammatory bowel diseases such as Crohn’s disease in humans [1].

Control of *paraTB* in livestock is complicated by the lack of appropriate tests for the identification of animals in the early phase of the disease. This is due to the low sensitivity of established direct and indirect diagnostic methods [2]. Although the initial infection usually takes place shortly after birth, clinical symptoms such as weight loss and diarrhea rarely appear until two or more years later. Likewise, fecal shedding of MAP and formation of specific antibodies are not detectable in the first years after infection. Concentrations of bacteria in feces increase only with progress of the disease [3]. A strong correlation between intensity of shedding and formation of specific antibodies has been noted and the highest proportion of antibody positive animals can be found in animals shedding high amounts of MAP [4].

In addition to the biological constraints, direct detection of MAP in feces has also methodological limitations. Detection of MAP genome by PCR can be accomplished within three days. However, the method is sensitive only in highly shedding animals. Cultivation of the bacteria from fecal samples is the most sensitive method but it is limited by their long replication time. Using solid media, between 4 and 18 weeks are necessary to obtain visible colony growth. Different automated liquid culture systems have been exploited to speed up cultivation of MAP. They either rely on the detection of bacterial metabolites in the headspace above the culture broth or within the broth itself [5–9], or on the consumption of gases by viable bacteria resulting in a negative pressure change within the headspace of the growth bottle [10]. Despite the reduction of cultivation time, the systems are not specific for the detection of mycobacteria and need subsequent confirmation of the presence of MAP by another test. Furthermore, they all are prone to contamination by other micro-organisms leading to non-specific signals.

Therefore, fast, cheap, and sensitive tests for the detection of MAP *in vivo* and *in vitro* would be desirable. Based on the knowledge that bacteria emit volatile organic compounds (VOCs) [11,12] a number of studies has been undertaken to identify potential volatile biomarkers for mycobacterial infections [13–22]. Differences between VOC patterns of infected and not infected animals were described in first pilot experiments applying less specific techniques such as electronic noses [13] and IMS [18]. Although these first results are promising, any detailed knowledge on potential marker compounds concerning their biochemical origin and their physico-chemical properties is lacking [18].

In the presented study, solid phase micro extraction and gas chromatography / mass spectrometry (SPME-GC-MS) were used to identify VOCs in the headspace of cultures containing different MAP strains. In detail, it was examined if characteristic volatile biomarkers could be detected in the headspace of MAP cultures in comparison to culture media and how the emission of VOCs was affected by density of bacterial growth.

## Materials and Methods

### Chemicals and Materials

#### Reference substances

Acetone, 2-butanone, hexanal, methacrolein, isoprene and benzene were obtained from Ionimed Analytik GmbH (Innsbruck, Austria). Butane, pentane, hexane and 2-ethoxy-2-methylpropane were bought from Supelco (Bellefonte, USA). Methyl isobutyl ketone, 3-octanone. 2-heptanone, 2-methylpropanol, 2-methylpentene, furan, 2-methylpropanal, heptane, methylacetate, ethyl acetate, 2-methylfuran, 2-ethylfuran, 2-pentylfuran, 4-methylheptane, dimethyldisulfide, 2-methylbutanenitril and 2-methyl-2-butenal came from Sigma-Aldrich (Steinheim, Germany). Octane was purchased from Merck KGaA (Darmstadt, Germany). 1-methylpyrrole, 2,4-dimethylheptene, 4-methyloctane, 3-methylfuran, 3-methylbutanal and 2,4-dimethylheptane was bought from TCI Europe N.V. (Zwijndrecht, Belgium).

SPME fibre assembly (PDMS-Carboxen 75µm was bought from Supelco (Bellefonte, USA).

#### MAP-Cultures

MAP strains were cultivated on Herrold’s egg yolk medium containing mycobactin J and amphotericin, nalidixic acid and vancomycin (HEYM-MJ, Becton Dickinson, Sparks, USA). Two loopfuls bacteria were suspended in 4 mL of Middlebrook 7H9 liquid medium containing oleic acid, albumin, dextrose, catalase, mycobactin J and polymyxin B, amphotericin B, carbenicillin and trimethoprim (MB). The bacterial suspension was thoroughly vortexed in the presence of sterile glass beads, bacterial clumps were allowed to settle and dilutions of 10°, 10-2, 10-4 and 10-6 were prepared from the supernatant in MB. Two hundred µL of the bacterial suspensions were inoculated onto each of two slopes of HEYM-MJ per dilution. The vials were sealed with silicone/Teflon septa. Cultures were incubated at 37°C in horizontal position for one week and then in upright position for five further weeks. Two vials of HEYM-MJ without bacteria served as control.

The following MAP strains were used:

• DSM 44133 (ATCC19689), laboratory adapted reference strain
• JIII-386, field strain from sheep
• JII-2421, field strain from cattle
• JII-3197, field strain from cattle
• JII-0817, field strain from red deer

Field strains were primary isolates from tissue (strain JIII-386 – ileum, JII-0817 - small intestine) or feces (strain JII-2421 and strain JII-3197), and have been isolated and cultivated according to standard protocols recommended by the national reference laboratory. Molecular genetic characteristics of the strains are shown in table 1. All in all 5 MAP strains, each with 4 serial dilutions, were measured in duplicates. Additionally two blank media samples were analysed.

**Table 1:** Molecular genetic characteristics of the investigated MAP strains.

			IS900-RFLP [51]	MIRU-VNTR [52]	MLSSR [53]
			BstI-PstII	Loci 292-X3-25-47-3-7-10-32	Loci 1-2-8-9
1	ATCC 19698	C-type^[40^	C5-P1	32332228	7-10-5-5
2	JIII-386	S-type^#^	I6-P13	421311*18	7-12-3-4
3	JII-2421	C-type	C1-P1	32332228	7-11-4-4
4	JII-3197	C-type	C1-P1	22522226	7-(-)-4-4
5	JII-0817	C-type	C17-P8	32332228	7-11-4-4

# Type III

### Sample Preparation

The headspace over the MAP cultures was pre-concentrated by means of Solid Phase Micro Extraction (SPME). A Carboxen/ PDMS fibre, 75 µm (Supelco, Belfonte PA) was used for all measurements. SPME fibres were pierced through the septum and exposed for 20 min to the headspace of the culture.

Before SPME-fibres were used for the first time, they were conditioned in the injector of a GC at 300°C for 1h. Afterwards the tip of the fibre was sealed with a Teflon cap until usage. Before reuse, SPME fibres were conditioned again for 30min. A blank run of the fiber was performed on every day before the measurements to ensure that the SPME coating was clean and that no uncontrolled bleeding took place. In order to control the SPME fiber quality, a gas standard was analysed at the beginning and at the end of every GC-MS sampling queue.

Before SPME fibres were exposed to the headspace, MAP cultures were conditioned at 37°C for 20min. This temperature was also held constant during sampling.

### GC-MS analysis

SPME fibres were thermally desorbed in the GC-injector at an injector temperature of 290°C. A 0.8 mm SPME inlet liner (Bellefonte, PA, USA) and splitless injection was applied. During sample injection the injector was operated in splitless mode for 60s at a carrier gas flow rate of 1.7 ml/min. After the injection phase a split ratio of 30 resulting in a total inlet flow rate of 50 ml/min was applied. This split ratio was maintained during the remaining analysis time.

An Agilent 7890A gas chromatograph connected to an Agilent 5975C inert XL mass selective detector (MSD) was used for separation and detection of volatile organic compounds desorbed from SPME-fibres. The GC was equipped with a CP-Pora Bond Q Fused Silica Column (Varian Inc, CA). The temperature program worked as follows: 90°C for 6 min, 15 °C/min to 120°C for 1 min, 10 °C/min to 140°C for 7 min, 15 °C/min to 260°C for 6 min.

Total ion current during mass spectrometric detection with electron impact ionisation (70 eV) was used. Mass range was 35-300 amu and the scan rate was 2.73 scan/s. The temperature of the ion source was 230°C, the temperature of the transfer line was 280°C.

### Calibrations, LODs and LOQs

Gas standards of the potential marker substances were prepared in concentrations between 0.46 and 1933.4 ppbV. For calibration and for determination of LOD and LOQ 10 different concentration levels were prepared.

Calibrations mixtures of pure reference materials were prepared by transferring liquid reference substances into a 100 mL evacuated gas sampling bulb by means of a 10µL syringe. The gas sampling bulb was then equilibrated with nitrogen. 50 µL of the gas mixture were transferred to a tedlar bag filled with 1 L of nitrogen. Different concentration levels were prepared by dilution with nitrogen. 15 mL from these gas standards were then transferred into evacuated 20 mL headspace vials and pressure was equilibrated with nitrogen. All gas standards were pre-concentrated by means of SPME under the same conditions (37°C, 20min) as the headspace of the MAP cultures.

LODs and LOQs for the SPME-GC-MS method were determined by means of the signal to noise ratio. Noise level was determined experimentally from blank samples. LOD was defined as S/N of 3, LOQ as S/N of 10.

## Results

### Identification and Quantitation of potential VOC marker substances

SPME-GC-MS measurements resulted in more than 100 single substances detectable in the headspace over the MAP cultures.

**Figure 1:**
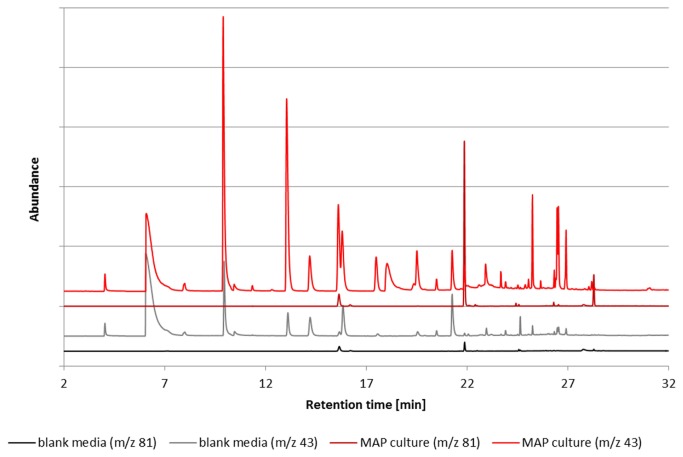
Chromatograms of selected ion traces (m/z 43 & m/z 81) from the headspace of the reference strain DSM 44133 (dilution of bacteria of 10^-2^; red chromatograms) and blank media (blue chromatograms). Retention times of marker substances are described in table 2.

To differentiate between volatile compounds from the material or the media and those originating from the MAP cultures we compared results from the cultures with those from the media. Only VOCs not detectable in the media or VOCs with significant higher concentrations in the different cultures compared to the media blank (> 120% compared to media) were considered as potential biomarkers. Altogether, 34 potential marker substances could be identified in the cultures and were investigated in more detail. Selected ion chromatograms from a SPME-GC-MS headspace measurement of a MAP culture and a blank medium are shown in Figure 1.

To identify those compounds we used a spectral library (NIST 2005 Gatesburg, PA, USA) in a first approach. In a second step GC retention times and mass spectra of the potential marker compounds were confirmed by means of pure reference substances.

Table 2 shows retention times and quantitative parameters (qualifier ions, LODs and LOQs) of all 34 verified substances.

**Table 2:** Substance identities as confirmed through retention time and mass spectra of reference substances.

**Substance**	**Retention time** (min)	**Quant Ion**(m/q)	**Slope**	**R^2^**	**LOD** (ppbV)	**LOQ** (ppbV)
2-Ethylfuran	21.95	81	12278	0.998	0.19	0.65
2-Methylfuran	15.82	82	8569.5	0.993	9.55	31.83
3-Methylfuran	16.62	82	8471.9	0.996	0.13	0.42
Furan	9.81	68	4624.9	0.999	0.10	0.35
2-Pentylfuran	28.40	81	649.56	0.995	4.64	15.45
2-Heptanone	26.46	43	1227	0.999	2.98	9.94
3-Octanone	28.36	43	244.29	0.999	22.64	75.46
Acetone	10.14	58	37285	0.998	16.96	56.53
Methyl Isobutyl Ketone	24.00	43	5989.9	0.998	1.26	4.19
2-Butanone	16.11	43	55243	0.999	1.43	4.75
3-Methyl-Butanal	21.50	44	1953.2	0.999	0.98	3.28
2-Methylpropanal	14.60	43	3050	0.999	0.25	0.83
Methacrolein	14.13	70	2727.2	0.999	0.12	0.41
2-Methylbutanal	21.58	57	2451.8	0.999	0.70	2.33
2-Methyl-2-Butenal	22.77	84	1688.2	0.997	0.26	0.86
Hexanal	24.71	56	15793	0.999	4.66	15.52
4-Methylheptane	25.30	43	6367.3	0.996	2.04	6.81
4-Methyloctane	26.98	43	3674.3	0.997	2.30	7.66
2,4-Dimethylheptane	26.60	43	2167.6	0.999	1.41	4.71
Butane	8.08	43	27544	0.999	3.48	11.60
Pentane	13.24	72	7153.3	0.993	5.55	18.49
Hexane	20.60	86	9507.2	0.993	7.27	24.23
Heptane	23.83	43	2651.3	0.998	2.64	8.81
Octane	25.79	43	1967.2	0.995	3.85	12.85
2,4-Dimethyl-1-Heptene	26.60	43	2534	0.98	19.19	63.98
2-Methyl-1-Pentene	19.80	56	5081.4	0.999	0.73	2.43
Isoprene	12.62	67	52400	0.999	2.14	7.14
Methylacetat	11.57	43	3207.4	0.999	0.29	0.97
Ethylacetate	17.93	43	4044.2	0.999	0.19	0.64
2-Ethoxy-2-Methylpropane	21.81	59	9559.3	0.999	0.43	1.43
1-Methyl-1-H-Pyrrole	22.32	81	2760.3	0.999	0.26	0.87
2-methylbutanenitril	22.97	55	7348.7	0.999	6.54	21.80
Dimethyldisulfide	22.61	94	4219.9	0.993	0.08	0.26
Benzene	20.20	78	163737	0.991	8.86	29.52

Calibration data and LOD/LOQ for the SPME-GC-MS assay

### VOC marker patterns in MAP cultures

**Figure 2:**
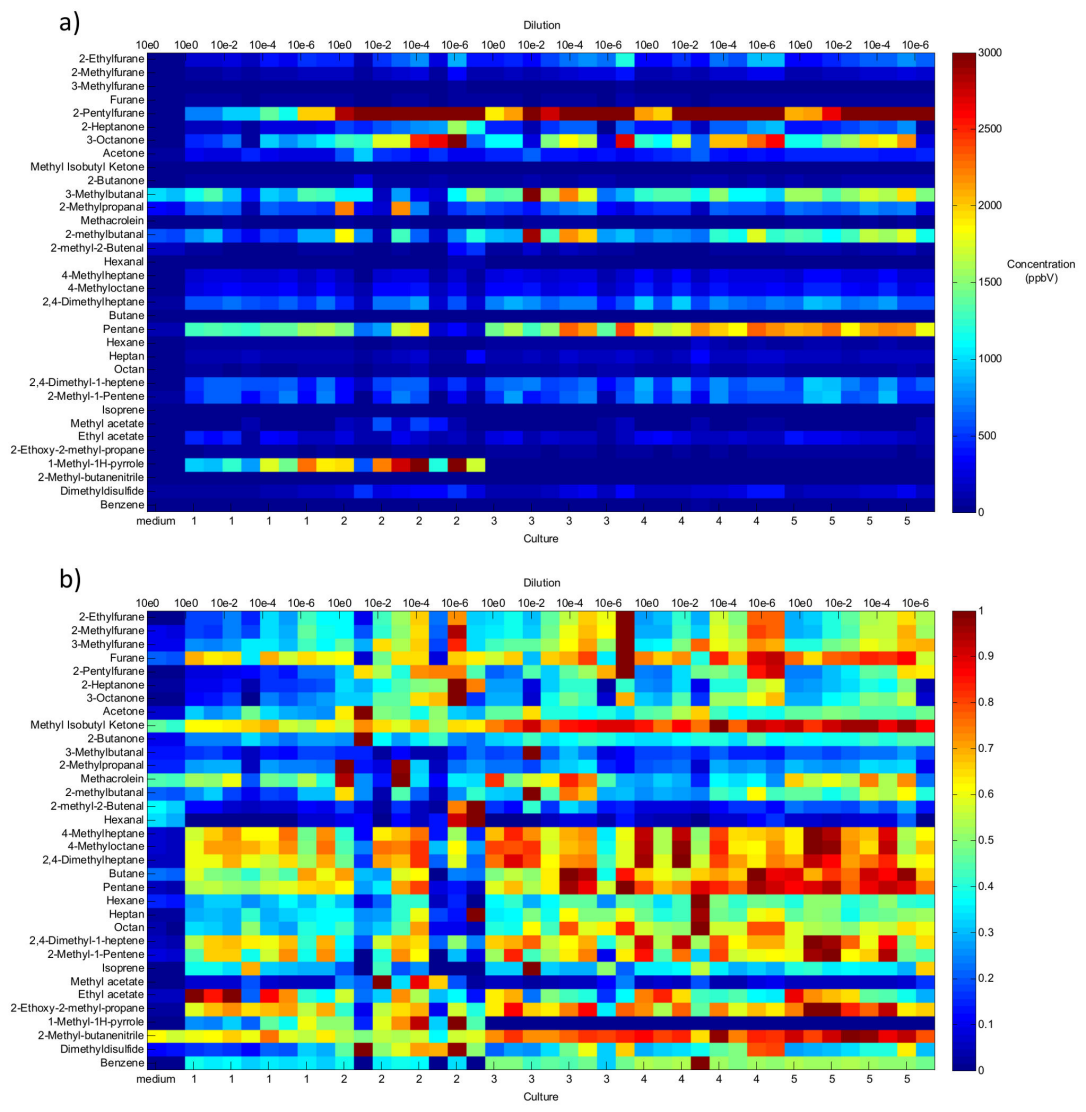
Heat-map with all selected VOCs from the five different MAP strains as well as from the media a) quantitative data b) normalized data; Cultures: 1=DSM 44133, 2=JIII-386, 3-5=JII-2421, JII-3197, JII-0817.

Figure 2 shows the VOC concentration patterns of all investigated MAP strains and dilutions as a heat map. The different VOCs are plotted on the y-axis, while the different MAP strains can be found on the x-axis with increasing dilution from left to right within each strain. Blank media and the five investigated MAP strains are separated by red lines. Figure 2a shows quantitative data (ppbV), while Figure 2b shows data normalized to the maximum concentrations determined in all samples. In this way, the variance between the samples is emphasized and relative differences can be seen.

Comprehensive quantitative data can be found in the supplement. Table S1 shows concentrations of all identified marker VOCs determined from the headspace of the mycobacterial cultures. These data refer to all mycobacterial strains and to all dilutions used in this study.

Among the identified potential marker substances, furans, aldehydes, aliphatic and unsaturated hydrocarbons and some nitrogen containing compounds were the most abundant.

### Furans

Among the detected substances substituted furans exhibited the highest concentrations over MAP cultures. Concentrations as high as 7 ppmV were found for 2-pentylfuran. Furan, 2-methylfuran and 3-methylfuran concentrations emitted from the cultures were 2-6 times higher than those emitted from the blank media, 2-ethylfuran and 2-pentylfuran concentrations were 15-191 times higher in the cultures than in the media.

### Aldehydes

3**-**methylbutanal, 2**-**methylbutanal, 2**-**methylpropanal and methacroleine concentrations were above LOQ in all MAP samples as well as in the blank media samples. All samples from the cattle field strains emitted (1.5-2 times) higher concentrations of 2**-**methylbutanal when compared to the blank media. 3**-**Methylpropanal concentrations were higher in all samples from the two cattle strains JII-0817 and JII-3197when compared to the media. The majority of cultures of the sheep strain showed lower concentrations than the media.

### Hydrocarbons

Pentane, 4-methylheptane, 4-methyloctane and 2,4-dimethylheptane concentrations were always higher in the MAP samples when compared to the media. However, most hydrocarbons were still detectable in the media. Heptane concentrations were below LOQ in the blank media samples and higher within all MAP samples. Octane concentrations were above LOQ in the MAP cultures. Hexane concentrations were lower in the sheep strain emissions and higher in all other samples when compared to the media.

2,4-dimethyl-1-heptene was below LOQ in the blank media samples and higher in the MAP samples. 2-methyl-1-pentene could be detected in blank media and in the cultures, however concentrations were higher in most MAP samples. Isoprene was not detectable in the blank media samples and the concentration was below LOQ for most MAP samples.

### N and S containing compounds

1-methyl-1-H-pyrrole could not be detected in the three cattle field strains, but in the reference and the sheep strain. 1-methyl-butanenitrile was higher in all samples from the cattle field strains when compared to the media.

Dimethyldisulfide concentrations were higher in MAP samples than in the media.

### Differentiation of MAP strains and media

With the exception of ethyl acetate, the laboratory adapted reference strain tended to exhibit lower concentrations of most substances, when compared to the other four MAP strains. Ethyl acetate concentrations seemed to decrease with higher dilutions.

In contrast, the sheep strain JIII-386 showed the largest variations between the different samples (replicates and dilutions), and also emitted the highest substance concentrations.

Concentrations of saturated and unsaturated hydrocarbons tended to be higher in the cattle field strains than in the other two MAP strains, while the highest amounts of aldehydes could be found in strain JIII-386, although the variance among the different samples was high.

Although all 34 selected compounds were emitted from almost all culture samples, relative abundances of the investigated compounds were different between the reference strain, the sheep and the cattle strains.

**Figure 3:**
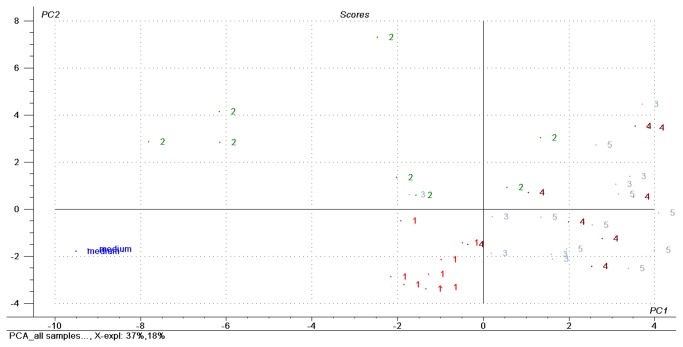
Principal component analysis on VOCs emitted from 5 different MAP strains and the blank media. 1: DSM 44133 (laboratory reference strain), 2: JIII-386 (sheep strain), 3-5: JII-2421, JII-3197, JII-0817 (field strains).

Figure 3 shows the score plot of a principal component (PCA) analysis including all 34 VOCs, with principal component 1 (PC 1) and PC 2 forming the coordinate system. PCA was performed using The Unscrambler 9.7 (Camo Software AS). Quantified data were used to calculate the PCA. Data were normalized onto standard deviation of the corresponding data set and centered onto mean values prior to the PCA. Cross validation was used as validation method. Principal component 1 (PC 1) explains 37% of the variance in the data, while PC 2 explains 18%.

The blank media measurements were negatively correlated on PC 1 and PC 2 and were clearly separated from all five MAP strains. The reference strain showed a negative correlation on PC 1 and PC 2, while the sheep strain was positively correlated on PC 2 and the cattle field strains were positively correlated on PC 1 with positive and negative correlations on PC 2. The high variations of measured VOC concentrations between the replicates and dilutions within the sheep strain were also reflected in the PCA results. While the three cattle field strains as well as the reference strain tended to form clusters in the score plot, the scores of the sheep strain were widely spread.

### Effects of bacterial density on VOC emissions

**Figure 4:**
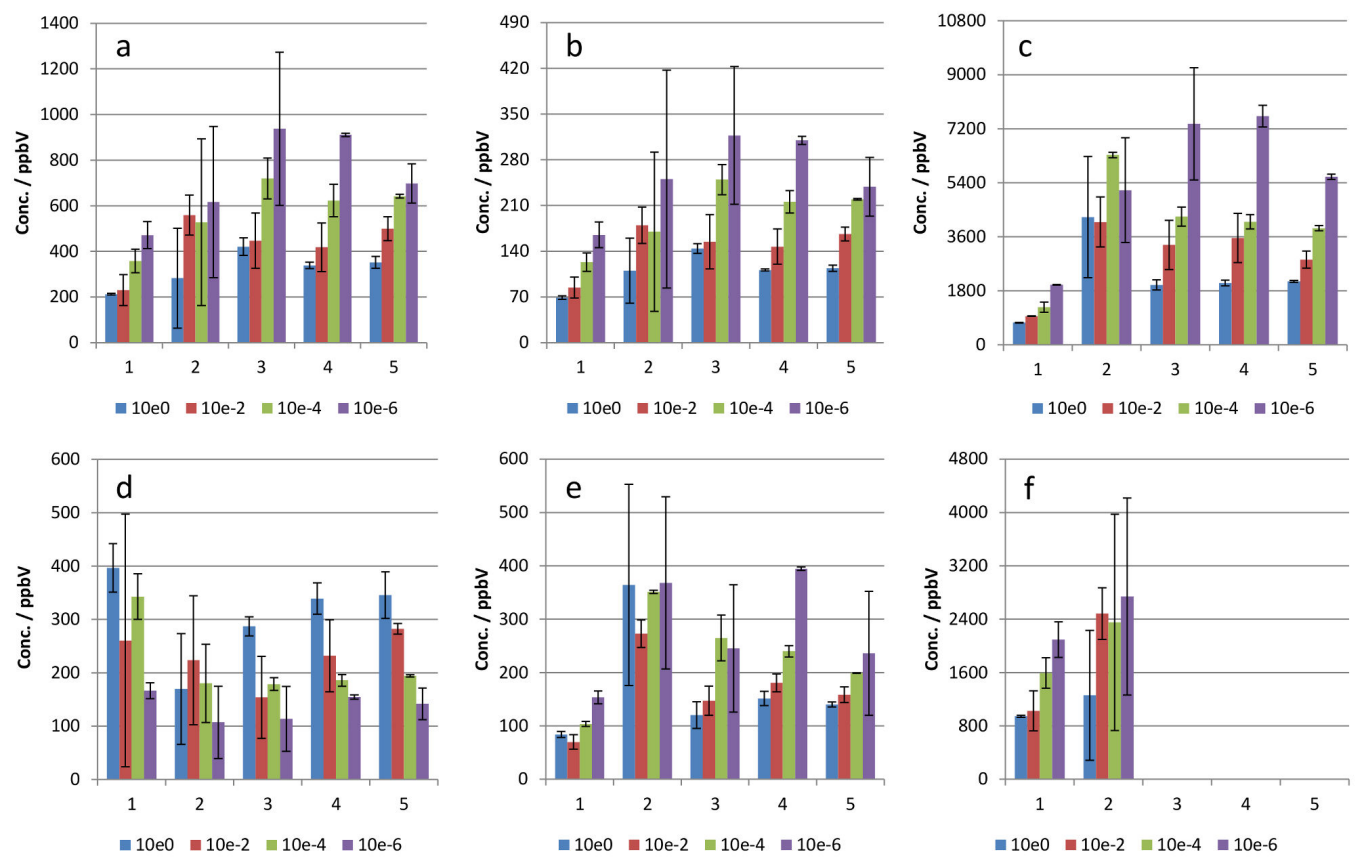
Effect of density of bacterial growth on emitted amounts of VOCs. **1: DSM 44133 (laboratory reference strain), 2: JIII-386 (sheep strain), 3-5: JII-2421, JII-3197, JII-0817 (field strains)**. a) 2-ethylfuran b) 2-methylfuran c) 2-pentylfuran d) ethyl acetate e) dimethyldisulfide f) 1-methyl-1-H-pyrrole.

Figure 4 illustrates the effect of different dilutions of the MAP strains onto the concentrations of 2-ethylfuran, 2-methylfuran, 3-methylfuran, 2-pentylfuran, ethyl acetate, 1-methyl-1-H-pyrrole and dimethyldisulfide.

Concentrations of (substituted) furans (Figure 4a-c) in the headspace over the cultures decreased with higher bacterial density. The effect was less pronounced in the sheep strain JIII-386. 3-Methylfuran showed a less pronounced concentration effect when compared to the other three substituted furans. Concentrations of furan did not show a dependency on bacterial density.

In contrast, emissions of ethyl acetate (Figure 4d) increased with increasing bacterial density

1-methyl-1-H-pyrrole (Figure 4f) was only detected in the reference and the sheep strain and showed a decrease with higher bacterial densities. Dimethyldisulfide (Figure 4e) showed a similar tendency as the substituted furans, but again, the effect was less pronounced within the intermediate strain.

## Discussion

More than 100 VOCs were detected in the headspace over *Mycobacterium avium* subsp. *paratuberculosis* cultures by means of HS-SPME-GC-MS. Among those 34 compounds could be attributed to mycobacterial growth. All five MAP strains could clearly be distinguished from blank culture media by means of emission patterns based on these 34 substances. In addition, patterns of volatiles emitted by the reference strain were significantly different from the field strains. Headspace concentrations of 2-ethylfuran, 2-methylfuran, 3-methylfuran, 2-pentylfuran, ethyl acetate, 1-methyl-1-H-pyrrole and dimethyldisulfide correlated with density of bacterial growth.

Given the large number and the chemical diversity of volatiles emitted from bacterial cultures [23,24] unequivocal identification and reliable quantification is mandatory when these volatiles or patterns built up from them are to be used as biomarkers for bacterial growth. In this study, substance identification did not solely rely on MS-database (e.g. NIST) search but was confirmed by analysis of pure reference substances. In addition, reliable quantification was achieved through calibration with reference materials. LOD and LLOQ were determined for all substances discussed in this study.

Emission patterns from blank media and those from the different MAP strains differed qualitatively and quantitatively from each other. I.e. MAP cultures emitted volatiles that could not be found in the headspace of the media at all, other compounds had significantly higher concentrations in the headspace over MAP cultures. Among these substances furans, aldehydes, aliphatic and unsaturated hydrocarbons and some nitrogen containing compounds were the most abundant.

Different furans have already been detected in various microorganisms [23,25], [26] [27], [28,29], and have been proposed as indicators of fungal growth in cereal grain [29]. 2-pentylfuran was detected in the headspace of *Aspergillus fumigatus* as well as in the breath of patients with *Aspergillus fumigatus* infections [30,31]. 2-methylfuran, 2-ethylfuran and 2-pentylfuran are also known as VOCs originating from rhizobacteria, however concentration levels were very low [32], while concentrations in MAP strains were as high as ppmV. As *Aspergillus* overgrowth could definitely be excluded in all analysed MAP cultures, high furan concentrations seemed to be specifically due to mycobacterial growth. In mycobacteria, surface glycolipids contain D-galactofuran [33] and arabinofuranosyl residues [34]. Furfural and furans can be derived from these pentose compounds. In addition, furans may possibly be generated through oxidative degradation of fatty acids or may occur along synthetic pathways using acetyl-CoA building blocks [23].

From these biochemical pathways one may conclude that furans indicate (myco)bacterial growth in terms of cell wall turn over. Substituted furans that can be generated from D-galactofuran or arabinofuranosyl residues being parts of mycobacterial surface structures may even specifically indicate the growth of distinct mycobacteria.

Cell wall structures account for many of the unique properties of *Mycobacteria* species, including resistance to bactericidal effects or functions of the host and low permeability to antibiotics. Compounds structurally related to mycolic acids are found in significant amounts in mycobacteria. These compounds include long chain acids, alcohols and also ketones [35]. Mycolic acids represent important components of the cell envelope of mycobacteria. When these long chain fatty acids are cleaved enzymatically or thermally various aldehydes and ketones may be generated [36,37]. In addition, aldehydes have been identified as intermediates in the biosynthesis of lipid metabolites of mycobacteria [38]. Further knowledge on mycolic acid metabolism and more experimental data are necessary to know whether some aldehydes may represent specific markers for mycobacterial growth.

Given the structure of mycolic acids, branched hydrocarbons may also be generated through oxidative or enzymatic cleavage of these long chain fatty acids.

Dimethyldisulfide is known to be generated from cysteine or methionine via transamination or disproportionation pathways [23].

Based on the comparison of whole genomes of MAP, two major lineages have been distinguished, a sheep lineage and a cattle lineage [39], historically designated “Sheep or S-type” and “Cattle or C-type”, respectively [40]. Using genotyping methods, the S-type strains can be further divided into two subtypes, Type I and Type III [41]. In addition to genotypic differences, strains of the two lineages exhibit phenotypic differences, such as growth rate [40,42,43], utilization of different iron metabolic pathways [44] and cytokine induction in macrophages [45,46].

To take into account the genetic variability of MAP, a laboratory adapted strain, an S-type field strain and three C-type field strains were investigated. It was possible to distinguish the reference and the S-type strain from C-type field strains by means of their volatile emission patterns. 1-methyl-1-H-pyrrole could only be detected over cultures of the reference and the S-type strain. Pyrrols represent basic components of heme complexes which can be found in protein sensor [47] and catalase/peroxidase [48] enzyme systems of the mycobacteria. The fact, that 1-methyl-1-H-pyrrole had detectable concentrations only in the laboratory adapted and the S-type field strain may hypothetically be attributed on the one hand to the (phylo)genetic differences between sheep and cattle strains and, on the other hand, to different long-term environmental conditions these bacterial strains were exposed to. Both conditions may have resulted in the expression of specific enzyme systems. Likewise, long-term in vitro passage of vaccine strains of MAP under different culture conditions influenced MAP genome diversity resulting in large tandem genomic duplications, deletions and transposable element activity. In combination with classical selective systematic subculture this led to fixation of specific MAP genomic alterations in some vaccine strain lineages which link the resulting attenuated phenotypes with deficiencies in high reactive oxygen species handling [49].

Emissions from the three C-type field strains did not show marked differences.

Varying densities of bacterial growth in the MAP cultures induced differences in emissions of substituted furans, ethylacetate, dimethyldisulfide and methylpyrrole. Lower growth densities resulted in higher concentrations of furans, dimethyldisulfide and methylpyrrol. Volatile substances, e.g. methyl-2,3,3,4-tetrahydroxyhydrofuran (S-THMF), have been described as signalling substances in cell to cell communication in gram-positive and gram-negative bacteria [12,50]. Hence, increasing concentrations of furans, dimethyldisulfide and methylpyrrole might represent chemical signals for the potential of further cell growth in cultures with low bacterial density. As ethylacetate is generated during the biosynthesis of fatty acids [23] higher quantities of growing cells will lead to higher concentrations of this substance in the headspace over the cultures.

Although results from all MAP culture dilutions were fed into the principal component analysis, separation of the laboratory adapted and the S-type strain from the C-type field strains was possible. Hence, emission patterns based on 34 selected VOCs not only provided separation of blank media from MAP cultures but also enabled distinction of different MAP strains regardless of bacterial growth density.

Analysis of VOCs emitted from mycobacterial cultures may be used to identify bacterial growth and differentiate between different bacterial strains. VOC emission patterns can provide valuable information on genetically and environmentally determined alterations of metabolic pathways. In addition, they may enable approximation of bacterial growth density.

In a perspective volatile marker substances could be used to diagnose MAP infections in animals and to identify different bacterial strains and origins.

## Supporting Information

Table S1 **Detected Headspace concentrations (ppbV) of all investigated MAP culture samples and blank media samples.** Blue lettering: detected amounts below LOQ; Yellow background: concentration in the same range as media concentration. Red background: concentration less than blank media concentration. No background color: concentration higher than blank media concentration. 1: reference strain, 2: intermediate strain, 3-5: field strains.
(DOCX)
