# Supplementary material for: Volatile Emissions from Mycobacterium avium subsp. paratuberculosis Mirror Bacterial Growth and Enable Distinction of Different Strains
Source: PLoS One. 2013 Oct 8;8(10):e76868. doi: 10.1371/journal.pone.0076868 (PMC3792893; doi:10.1371/journal.pone.0076868)
Supplement: Table S1 — Detected Headspace concentrations (ppbV) of all investigated MAP culture samples and blank media samples. Blue lettering: detected amounts below LOQ; Yellow background: concentration in the same range as media concentration. Red background: concentration less than blank media concentration. No background color: concentration higher than blank media concentration. 1: reference strain, 2: intermediate strain, 3-5: field strains. (DOCX) [file pone.0076868.s001.docx]

| **Substance** | **Media A** | **Media B** | **1 A 10e^0^** | **1 B 10e^0^** | **1 A 10e^-2^** | **1 B 10e^-2^** | **1 A 10e^-4^** | **1 B 10e^-4^** | **1 A 10e-^6^** | **1 B 10e^-6^** |
| --- | --- | --- | --- | --- | --- | --- | --- | --- | --- | --- |
| 2-Ethylfuran | 14.20 | 13.04 | 214.88 | 209.91 | 278.27 | 182.98 | 394.47 | 321.58 | 513.25 | 429.49 |
| 2-Methylfuran | 38.95 | 36.45 | 70.91 | 67.18 | 95.63 | 73.13 | 133.17 | 113.30 | 179.01 | 151.21 |
| 3-Methylfuran | 5.86 | 5.39 | 14.86 | 13.97 | 15.43 | 20.16 | 20.59 | 17.44 | 24.27 | 20.83 |
| Furan | 20.41 | 18.14 | 66.48 | 61.73 | 62.26 | 34.19 | 66.63 | 54.82 | 63.21 | 58.60 |
| 2-Pentylfuran | 46.10 | 39.60 | 728.72 | 743.44 | 949.16 | 959.63 | 1376.92 | 1137.99 | 1993.15 | 2006.00 |
| 2-Heptanone | 34.44 | 28.95 | 155.49 | 170.83 | 206.66 | 72.63 | 250.44 | 246.90 | 278.24 | 311.67 |
| 3-Octanone | 0.00 | 0.00 | 466.22 | 496.88 | 688.67 | 55.29 | 939.90 | 772.59 | 910.57 | 1031.65 |
| Acetone | 64.54 | 59.63 | 302.25 | 251.45 | 279.83 | 461.30 | 313.92 | 269.61 | 262.25 | 243.13 |
| Methyl Isobutyl Ketone | 15.45 | 14.16 | 20.55 | 20.95 | 21.37 | 22.10 | 23.48 | 19.40 | 18.38 | 21.13 |
| 2-Butanone | 26.36 | 24.00 | 58.61 | 61.01 | 60.76 | 83.29 | 65.39 | 56.22 | 65.37 | 65.37 |
| 3-Methylbutanal | 1007.27 | 931.78 | 1132.91 | 1326.54 | 931.02 | 382.62 | 980.75 | 885.49 | 1348.54 | 1306.93 |
| 2-Methylpropanal | 332.60 | 317.13 | 624.63 | 658.85 | 649.65 | 129.26 | 609.64 | 547.43 | 542.93 | 510.32 |
| Methacrolein | 36.86 | 36.64 | 44.57 | 42.21 | 50.69 | 16.09 | 39.82 | 37.77 | 45.51 | 33.77 |
| 2-methylbutanal | 598.27 | 552.02 | 756.47 | 923.80 | 508.76 | 309.47 | 532.51 | 491.24 | 884.50 | 871.79 |
| 2-methyl-2-Butenal | 179.29 | 161.44 | 61.10 | 60.12 | 38.17 | 23.36 | 35.28 | 31.44 | 56.69 | 51.15 |
| Hexanal | 37.54 | 32.49 | 0.00 | 0.00 | 0.00 | 0.00 | 0.00 | 0.00 | 7.49 | 7.44 |
| 4-Methylheptane | 23.38 | 19.11 | 206.08 | 243.79 | 262.98 | 227.67 | 224.38 | 277.13 | 172.30 | 262.90 |
| 4-Methyloctane | 30.31 | 26.93 | 275.76 | 325.77 | 331.68 | 320.39 | 296.30 | 332.29 | 224.93 | 346.50 |
| 2,4-Dimethylheptane | 56.53 | 47.18 | 582.98 | 582.42 | 685.64 | 594.33 | 549.50 | 632.73 | 511.79 | 688.65 |
| Butane | 5.80 | 5.33 | 15.29 | 12.74 | 12.31 | 10.87 | 13.25 | 11.70 | 18.68 | 15.79 |
| Pentane | 122.33 | 101.54 | 1292.59 | 1319.86 | 1266.86 | 1228.40 | 1362.56 | 1403.82 | 1566.32 | 1609.56 |
| Hexane | 28.27 | 25.36 | 53.95 | 62.24 | 62.99 | 51.80 | 61.31 | 70.56 | 59.56 | 74.50 |
| Heptan | 5.95 | 5.75 | 101.93 | 105.35 | 101.10 | 146.50 | 102.35 | 110.87 | 121.13 | 130.07 |
| Octan | 6.02 | 0.00 | 50.33 | 49.20 | 44.38 | 77.54 | 44.79 | 40.47 | 60.74 | 55.63 |
| 2,4-Dimethyl-1-heptene | 46.06 | 41.83 | 514.77 | 625.58 | 650.63 | 602.23 | 569.19 | 643.75 | 396.93 | 668.35 |
| 2-Methyl-1-Pentene | 84.10 | 72.19 | 434.04 | 634.26 | 636.72 | 371.23 | 553.97 | 704.09 | 369.39 | 705.46 |
| Isoprene | 0.00 | 0.00 | 5.25 | 5.08 | 4.60 | 9.31 | 4.34 | 4.06 | 3.83 | 3.97 |
| Methyl acetate | 3.76 | 2.87 | 39.09 | 41.24 | 40.73 | 119.17 | 40.93 | 34.29 | 36.17 | 33.79 |
| Ethyl acetate | 26.15 | 23.67 | 428.75 | 364.50 | 427.90 | 93.14 | 372.92 | 312.59 | 177.10 | 155.76 |
| 2-ethoxy-2-methyl-propane | 2.05 | 1.62 | 42.82 | 50.62 | 53.34 | 47.61 | 50.70 | 52.70 | 40.78 | 51.09 |
| 1H-Pyrrole, 1-methyl- | 0.00 | 1.57 | 955.02 | 933.67 | 1237.76 | 814.26 | 1754.61 | 1430.63 | 2283.73 | 1906.76 |
| 2-methyl-butanenitrile | 21.86 | 20.68 | 21.71 | 22.20 | 23.72 | 21.29 | 27.43 | 21.10 | 19.90 | 22.51 |
| Dimethyldisulfide | 58.67 | 64.21 | 80.11 | 87.95 | 79.65 | 60.26 | 106.96 | 100.46 | 161.88 | 145.02 |
| Benzene | 0.00 | 0.00 | 19.54 | 18.20 | 18.45 | 15.99 | 18.34 | 17.20 | 17.77 | 16.71 |

**Table S1** Detected Headspace concentrations (ppbV) of all investigated MAP culture samples and blank media samples. Blue lettering: detected amounts below LOQ; Yellow background: concentration in the same range as media concentration. Red background: concentration less than blank media concentration. No background color: concentration higher than blank media concentration. 1: reference strain, 2: intermediate strain, 3-5 field strains

| **Substance** | **2 A 10e^0^** | **2 B 10e^0^** | **2 A 10e^-2^** | **2 B 10e^-2^** | **2A 10e^-4^** | **2 B 10e^-4^** | **2 A 10e^-6^** | **2 B 10e^-6^** |
| --- | --- | --- | --- | --- | --- | --- | --- | --- |
| 2-Ethylfuran | 437.46 | 128.00 | 496.61 | 621.32 | 786.51 | 269.65 | 850.87 | 381.91 |
| 2-Methylfuran | 145.53 | 75.09 | 159.97 | 199.41 | 255.98 | 83.89 | 368.39 | 132.46 |
| 3-Methylfuran | 26.25 | 16.29 | 37.13 | 30.83 | 38.16 | 10.51 | 47.84 | 19.46 |
| Furan | 60.30 | 6.07 | 45.79 | 61.97 | 63.31 | 7.86 | 64.43 | 60.90 |
| 2-Pentylfuran | 2829.54 | 5683.31 | 4682.54 | 3505.84 | 6265.43 | 6388.28 | 6388.29 | 3915.49 |
| 2-Heptanone | 534.31 | 456.88 | 576.69 | 701.68 | 769.26 | 805.06 | 1562.03 | 1168.25 |
| 3-Octanone | 1271.39 | 1243.71 | 1771.57 | 1771.78 | 2485.44 | 2647.67 | 3878.00 | 611.34 |
| Acetone | 596.73 | 952.52 | 470.29 | 448.11 | 370.10 | 486.78 | 305.26 | 281.77 |
| Methyl Isobutyl Ketone | 19.01 | 24.28 | 21.90 | 19.85 | 22.87 | 17.60 | 21.78 | 21.24 |
| 2-Butanone | 64.80 | 245.33 | 90.82 | 68.34 | 84.96 | 107.06 | 65.00 | 56.40 |
| 3-Methylbutanal | 1032.95 | 1052.90 | 173.68 | 826.57 | 324.13 | 230.72 | 1122.61 | 1588.33 |
| 2-Methylpropanal | 2229.14 | 301.12 | 191.19 | 2159.65 | 719.72 | 118.01 | 513.20 | 620.23 |
| Methacrolein | 79.85 | 21.08 | 11.70 | 84.09 | 28.53 | 9.83 | 29.62 | 28.63 |
| 2-methylbutanal | 1859.68 | 754.21 | 119.99 | 1304.69 | 628.76 | 141.63 | 738.92 | 1183.26 |
| 2-methyl-2-Butenal | 90.33 | 40.00 | 12.45 | 83.30 | 30.55 | 13.40 | 366.13 | 498.07 |
| Hexanal | 10.99 | 12.61 | 0.00 | 13.96 | 6.18 | 15.57 | 104.54 | 114.05 |
| 4-Methylheptane | 151.39 | 43.35 | 231.73 | 249.08 | 276.41 | 50.31 | 191.42 | 55.52 |
| 4-Methyloctane | 204.05 | 111.00 | 349.76 | 344.66 | 381.86 | 140.72 | 282.77 | 128.11 |
| 2,4-Dimethylheptane | 466.48 | 160.28 | 697.05 | 668.23 | 801.19 | 205.02 | 579.17 | 202.59 |
| Butane | 18.60 | 8.00 | 7.32 | 15.30 | 15.97 | 0.00 | 6.21 | 5.72 |
| Pentane | 1483.40 | 675.03 | 836.17 | 1712.23 | 1934.38 | 208.86 | 335.98 | 145.82 |
| Hexane | 59.07 | 16.86 | 41.59 | 80.71 | 80.94 | 11.41 | 27.74 | 0.00 |
| Heptan | 126.39 | 90.06 | 97.20 | 144.99 | 210.36 | 63.36 | 47.51 | 350.71 |
| Octan | 65.64 | 41.44 | 47.33 | 68.04 | 106.51 | 15.26 | 15.96 | 6.99 |
| 2,4-Dimethyl-1-heptene | 355.11 | 185.16 | 549.41 | 639.71 | 611.04 | 203.44 | 480.96 | 234.21 |
| 2-Methyl-1-Pentene | 335.27 | 0.00 | 381.86 | 703.14 | 592.98 | 0.00 | 286.07 | 83.35 |
| Isoprene | 2.62 | 0.00 | 5.70 | 2.63 | 3.56 | 0.00 | 0.00 | 0.00 |
| Methyl acetate | 152.39 | 123.21 | 602.81 | 204.09 | 536.47 | 396.67 | 119.66 | 17.27 |
| Ethyl acetate | 243.10 | 96.45 | 138.00 | 309.05 | 232.18 | 128.58 | 154.97 | 59.47 |
| 2-ethoxy-2-methyl-propane | 30.96 | 7.34 | 40.84 | 48.34 | 51.99 | 7.43 | 42.01 | 28.92 |
| 1H-Pyrrole, 1-methyl- | 1948.10 | 569.37 | 2210.00 | 2757.53 | 3497.12 | 1205.89 | 3784.76 | 1696.18 |
| 2-methyl-butanenitrile | 19.45 | 17.53 | 19.71 | 19.30 | 20.23 | 13.41 | 20.51 | 21.01 |
| Dimethyldisulfide | 231.04 | 497.45 | 254.58 | 291.07 | 353.49 | 349.23 | 482.25 | 254.06 |
| Benzene | 19.37 | 0.00 | 18.32 | 19.19 | 18.84 | 0.00 | 17.18 | 0.00 |

| **Substance** | **3 A 10e^0^** | **3 B 10e^0^** | **3 A 10e^-2^** | **3 B 10e^-2^** | **3 A 10e^-4^** | **3 B 10e^-4^** | **3 A 10e^-6^** | **3 B 10e^-6^** |
| --- | --- | --- | --- | --- | --- | --- | --- | --- |
| 2-Ethylfuran | 393.20 | 447.75 | 361.54 | 533.00 | 656.49 | 782.97 | 700.32 | 1174.67 |
| 2-Methylfuran | 138.95 | 149.48 | 125.15 | 183.91 | 233.12 | 265.97 | 242.78 | 391.88 |
| 3-Methylfuran | 24.35 | 26.35 | 26.80 | 29.95 | 35.30 | 40.80 | 28.89 | 57.32 |
| Furan | 54.97 | 63.48 | 29.71 | 63.42 | 67.38 | 77.15 | 33.94 | 96.61 |
| 2-Pentylfuran | 1875.34 | 2110.45 | 3908.20 | 2746.79 | 4047.02 | 4499.68 | 6041.36 | 8685.84 |
| 2-Heptanone | 430.43 | 424.57 | 132.43 | 494.33 | 651.20 | 649.49 | 37.94 | 636.31 |
| 3-Octanone | 1109.78 | 1122.47 | 277.87 | 1463.10 | 1873.17 | 1654.38 | 250.92 | 2695.83 |
| Acetone | 432.10 | 433.26 | 622.49 | 386.52 | 380.70 | 346.12 | 381.20 | 324.20 |
| Methyl Isobutyl Ketone | 25.23 | 27.11 | 30.56 | 25.32 | 28.59 | 28.99 | 30.06 | 30.38 |
| 2-Butanone | 77.35 | 83.24 | 105.70 | 82.91 | 91.45 | 100.64 | 96.04 | 86.85 |
| 3-Methylbutanal | 1344.04 | 1369.91 | 7234.71 | 1582.01 | 2212.73 | 1770.56 | 658.21 | 854.57 |
| 2-Methylpropanal | 518.72 | 498.44 | 384.45 | 646.57 | 707.02 | 773.23 | 340.01 | 258.81 |
| Methacrolein | 69.30 | 40.53 | 47.32 | 53.33 | 70.34 | 62.88 | 36.80 | 24.31 |
| 2-methylbutanal | 935.00 | 871.08 | 2885.21 | 1301.20 | 2198.33 | 1975.25 | 834.07 | 892.64 |
| 2-methyl-2-Butenal | 81.49 | 61.16 | 205.84 | 84.02 | 168.36 | 136.66 | 63.88 | 75.03 |
| Hexanal | 0.00 | 0.00 | 0.00 | 5.76 | 9.33 | 9.93 | 5.11 | 15.02 |
| 4-Methylheptane | 250.45 | 303.76 | 266.57 | 211.20 | 256.09 | 264.35 | 127.52 | 217.51 |
| 4-Methyloctane | 368.91 | 365.28 | 380.22 | 261.45 | 312.88 | 330.68 | 173.97 | 273.72 |
| 2,4-Dimethylheptane | 741.87 | 879.62 | 796.10 | 612.51 | 711.96 | 725.17 | 416.45 | 572.51 |
| Butane | 12.85 | 14.32 | 13.05 | 16.41 | 26.08 | 24.68 | 10.19 | 22.10 |
| Pentane | 1407.46 | 1631.22 | 1279.93 | 1542.84 | 2309.65 | 2139.63 | 1380.23 | 2409.28 |
| Hexane | 73.15 | 81.44 | 56.57 | 73.52 | 90.63 | 90.21 | 49.95 | 88.99 |
| Heptan | 125.55 | 128.64 | 171.03 | 131.99 | 217.13 | 171.93 | 171.15 | 212.76 |
| Octan | 59.66 | 57.43 | 93.72 | 64.14 | 119.65 | 89.12 | 94.83 | 126.73 |
| 2,4-Dimethyl-1-heptene | 626.78 | 660.41 | 736.63 | 535.21 | 593.97 | 657.54 | 372.23 | 528.88 |
| 2-Methyl-1-Pentene | 499.34 | 802.42 | 414.08 | 502.13 | 611.56 | 716.50 | 101.14 | 609.16 |
| Isoprene | 4.33 | 4.41 | 13.46 | 4.25 | 3.84 | 3.92 | 6.75 | 2.79 |
| Methyl acetate | 33.19 | 37.35 | 13.60 | 27.07 | 35.72 | 34.34 | 70.22 | 145.03 |
| Ethyl acetate | 274.31 | 299.77 | 99.69 | 208.25 | 187.24 | 170.48 | 70.77 | 156.68 |
| 2-ethoxy-2-methyl-propane | 47.34 | 64.64 | 49.62 | 46.35 | 54.42 | 64.27 | 25.99 | 56.81 |
| 1H-Pyrrole, 1-methyl- | 0.00 | 0.00 | 0.00 | 0.00 | 0.00 | 0.00 | 0.00 | 0.00 |
| 2-methyl-butanenitrile | 25.90 | 29.14 | 26.32 | 25.83 | 27.52 | 29.44 | 28.70 | 30.03 |
| Dimethyldisulfide | 102.61 | 137.96 | 127.83 | 166.79 | 234.45 | 295.49 | 160.87 | 329.71 |
| Benzene | 25.02 | 25.22 | 22.23 | 23.31 | 23.06 | 24.23 | 16.42 | 24.47 |

| **Substance** | **4 A 10e^0^** | **4 B 10e^0^** | **4 A 10e^-2^** | **4 B 10e^-2^** | **4 A 10e^-4^** | **4 B 10e^-4^** | **4 A 10e^-6^** | **4 B 10e^-6^** |
| --- | --- | --- | --- | --- | --- | --- | --- | --- |
| 2-Ethylfuran | 328.99 | 348.92 | 493.90 | 342.73 | 673.30 | 573.29 | 916.27 | 905.59 |
| 2-Methylfuran | 110.29 | 112.44 | 166.26 | 127.90 | 227.72 | 203.59 | 313.91 | 305.25 |
| 3-Methylfuran | 19.82 | 21.34 | 28.20 | 43.90 | 34.51 | 31.46 | 42.07 | 39.55 |
| Furan | 71.01 | 63.46 | 70.30 | 38.87 | 80.25 | 64.98 | 86.89 | 89.50 |
| 2-Pentylfuran | 2126.82 | 1991.48 | 2979.65 | 4136.36 | 4267.14 | 3929.21 | 7365.47 | 7873.44 |
| 2-Heptanone | 391.06 | 389.23 | 518.92 | 155.58 | 618.45 | 633.77 | 838.22 | 908.75 |
| 3-Octanone | 1265.23 | 1121.03 | 1734.70 | 637.14 | 2046.74 | 2040.62 | 2308.16 | 2624.59 |
| Acetone | 418.65 | 436.67 | 514.99 | 634.08 | 413.72 | 351.67 | 377.56 | 359.81 |
| Methyl Isobutyl Ketone | 28.59 | 25.52 | 29.29 | 25.63 | 33.64 | 25.85 | 31.53 | 30.22 |
| 2-Butanone | 82.07 | 81.86 | 89.51 | 98.06 | 96.89 | 83.89 | 110.47 | 90.35 |
| 3-Methylbutanal | 1244.36 | 1352.85 | 1357.53 | 1128.42 | 1521.70 | 1082.97 | 1123.33 | 961.25 |
| 2-Methylpropanal | 439.85 | 478.33 | 499.77 | 171.52 | 628.75 | 566.18 | 606.52 | 500.34 |
| Methacrolein | 23.16 | 26.54 | 23.92 | 12.72 | 30.33 | 27.74 | 30.52 | 21.99 |
| 2-methylbutanal | 735.00 | 827.46 | 767.05 | 695.79 | 1245.51 | 1136.38 | 1780.47 | 1229.22 |
| 2-methyl-2-Butenal | 43.73 | 46.49 | 39.14 | 33.50 | 59.19 | 82.49 | 81.43 | 58.07 |
| Hexanal | 5.49 | 5.03 | 5.13 | 0.00 | 8.66 | 8.42 | 14.06 | 19.50 |
| 4-Methylheptane | 341.18 | 195.35 | 337.18 | 182.80 | 295.37 | 237.02 | 241.21 | 255.00 |
| 4-Methyloctane | 438.28 | 246.47 | 450.62 | 239.89 | 382.77 | 289.73 | 288.22 | 336.60 |
| 2,4-Dimethylheptane | 942.27 | 582.21 | 980.84 | 562.25 | 796.78 | 697.03 | 615.41 | 725.72 |
| Butane | 17.41 | 15.59 | 14.54 | 17.28 | 18.48 | 17.36 | 25.86 | 22.84 |
| Pentane | 1951.84 | 1647.71 | 1757.50 | 2181.21 | 1996.89 | 1849.87 | 2316.57 | 2195.86 |
| Hexane | 88.31 | 64.82 | 84.40 | 196.23 | 95.67 | 81.54 | 94.22 | 90.36 |
| Heptan | 168.65 | 137.90 | 151.63 | 336.06 | 165.98 | 155.31 | 212.88 | 219.62 |
| Octan | 86.62 | 79.37 | 77.03 | 150.54 | 86.32 | 86.30 | 118.97 | 118.59 |
| 2,4-Dimethyl-1-heptene | 866.39 | 507.39 | 887.58 | 499.84 | 753.50 | 590.75 | 658.97 | 664.89 |
| 2-Methyl-1-Pentene | 811.46 | 424.12 | 722.56 | 312.39 | 771.65 | 547.58 | 584.00 | 586.89 |
| Isoprene | 4.77 | 4.80 | 4.96 | 7.40 | 4.75 | 3.94 | 4.23 | 3.96 |
| Methyl acetate | 36.07 | 38.38 | 34.29 | 121.14 | 26.75 | 28.20 | 54.10 | 59.05 |
| Ethyl acetate | 318.33 | 359.73 | 279.55 | 184.05 | 178.22 | 193.76 | 157.77 | 151.71 |
| 2-ethoxy-2-methyl-propane | 54.32 | 39.88 | 56.78 | 34.46 | 54.81 | 43.67 | 50.23 | 47.11 |
| 1H-Pyrrole, 1-methyl- | 0.00 | 0.00 | 0.00 | 0.00 | 0.00 | 0.00 | 0.00 | 0.00 |
| 2-methyl-butanenitrile | 31.28 | 28.74 | 30.11 | 23.21 | 34.77 | 26.63 | 30.23 | 29.58 |
| Dimethyldisulfide | 141.93 | 161.01 | 168.79 | 192.58 | 232.59 | 247.46 | 392.16 | 396.78 |
| Benzene | 27.61 | 26.47 | 27.71 | 51.14 | 26.47 | 25.37 | 27.14 | 25.99 |

| **Substance** | **5 A 10e^0^** | **5 B 10e^0^** | **5 A 10e^-2^** | **5 B 10e^-2^** | **5 A 10e^-4^** | **5 B 10e^-4^** | **5 A 10e^-6^** | **5 B 10e^-6^** |
| --- | --- | --- | --- | --- | --- | --- | --- | --- |
| 2-Ethylfuran | 333.12 | 370.74 | 462.76 | 536.73 | 636.68 | 648.21 | 758.66 | 637.24 |
| 2-Methylfuran | 110.68 | 117.44 | 158.78 | 173.96 | 220.36 | 218.52 | 270.45 | 206.80 |
| 3-Methylfuran | 23.04 | 22.53 | 27.14 | 29.17 | 34.78 | 32.54 | 38.81 | 40.23 |
| Furan | 75.00 | 63.11 | 75.01 | 76.45 | 80.49 | 79.34 | 83.36 | 54.52 |
| 2-Pentylfuran | 2095.70 | 2137.70 | 2637.40 | 3038.58 | 3947.02 | 3823.54 | 5656.75 | 5540.01 |
| 2-Heptanone | 396.31 | 421.67 | 464.21 | 519.11 | 613.71 | 577.27 | 779.09 | 56.61 |
| 3-Octanone | 1145.97 | 1131.42 | 1445.46 | 1566.16 | 1934.88 | 1819.54 | 2177.34 | 259.67 |
| Acetone | 444.97 | 441.04 | 461.70 | 456.14 | 429.07 | 418.09 | 401.96 | 459.03 |
| Methyl Isobutyl Ketone | 31.46 | 27.60 | 30.79 | 32.36 | 32.57 | 29.74 | 32.28 | 29.17 |
| 2-Butanone | 95.29 | 89.42 | 92.30 | 94.18 | 105.02 | 99.53 | 107.65 | 108.73 |
| 3-Methylbutanal | 1567.87 | 1568.31 | 1326.87 | 1533.70 | 1689.62 | 1628.70 | 1939.63 | 1460.15 |
| 2-Methylpropanal | 665.21 | 622.85 | 589.04 | 613.99 | 750.13 | 702.35 | 743.16 | 243.04 |
| Methacrolein | 55.50 | 47.05 | 50.31 | 44.91 | 61.76 | 44.82 | 61.06 | 19.34 |
| 2-methylbutanal | 1313.66 | 1312.36 | 1107.28 | 1275.84 | 1663.75 | 1561.25 | 1771.91 | 1161.02 |
| 2-methyl-2-Butenal | 112.75 | 93.78 | 95.42 | 86.99 | 120.34 | 97.93 | 143.93 | 62.64 |
| Hexanal | 7.82 | 7.21 | 7.08 | 8.33 | 9.34 | 9.38 | 23.79 | 0.00 |
| 4-Methylheptane | 229.99 | 365.24 | 344.07 | 258.88 | 245.49 | 328.35 | 194.84 | 227.44 |
| 4-Methyloctane | 289.01 | 461.69 | 453.81 | 334.28 | 301.81 | 429.13 | 241.73 | 310.90 |
| 2,4-Dimethylheptane | 690.33 | 916.04 | 853.92 | 691.16 | 735.88 | 817.92 | 571.25 | 604.38 |
| Butane | 23.49 | 18.10 | 21.38 | 19.67 | 23.68 | 22.63 | 25.60 | 17.26 |
| Pentane | 2106.26 | 2147.69 | 2278.43 | 1869.69 | 2133.41 | 2227.04 | 2175.04 | 1821.80 |
| Hexane | 78.17 | 99.66 | 99.51 | 78.27 | 86.40 | 99.59 | 82.46 | 77.38 |
| Heptan | 163.00 | 180.25 | 169.90 | 136.14 | 161.26 | 164.49 | 179.18 | 185.34 |
| Octan | 87.87 | 80.87 | 79.51 | 73.88 | 87.26 | 82.77 | 96.56 | 91.64 |
| 2,4-Dimethyl-1-heptene | 552.26 | 951.99 | 912.52 | 737.04 | 572.16 | 829.09 | 470.81 | 623.90 |
| 2-Methyl-1-Pentene | 579.76 | 919.04 | 961.68 | 600.49 | 637.70 | 911.51 | 463.89 | 425.67 |
| Isoprene | 5.39 | 5.56 | 4.80 | 5.03 | 4.73 | 4.80 | 3.87 | 9.03 |
| Methyl acetate | 35.73 | 37.47 | 32.41 | 31.78 | 30.39 | 29.34 | 38.68 | 94.69 |
| Ethyl acetate | 376.37 | 314.79 | 289.52 | 275.42 | 195.84 | 192.64 | 162.75 | 120.86 |
| 2-ethoxy-2-methyl-propane | 55.50 | 73.46 | 74.04 | 61.95 | 57.61 | 69.12 | 46.54 | 48.78 |
| 1H-Pyrrole, 1-methyl- | 0.00 | 0.00 | 0.00 | 0.00 | 0.00 | 0.00 | 0.00 | 0.00 |
| 2-methyl-butanenitrile | 32.98 | 28.81 | 32.61 | 35.84 | 35.22 | 31.86 | 33.25 | 28.45 |
| Dimethyldisulfide | 136.77 | 143.49 | 147.83 | 168.70 | 199.49 | 198.92 | 318.13 | 154.06 |
| Benzene | 27.84 | 26.45 | 27.17 | 27.80 | 26.58 | 25.82 | 25.91 | 24.12 |
